# Marked Efficiency Improvement of FAPb_0.7_Sn_0.3_Br_3_ Perovskite Light-Emitting Diodes by Optimization of the Light-Emitting Layer and Hole-Transport Layer

**DOI:** 10.3390/nano12091454

**Published:** 2022-04-25

**Authors:** Lufeng Hu, Zhixiang Ye, Dan Wu, Zhaojin Wang, Weigao Wang, Kai Wang, Xiangqian Cui, Ning Wang, Hongyu An, Bobo Li, Bingxi Xiang, Mingxia Qiu

**Affiliations:** 1 College of New Materials and New Energies, Shenzhen Technology University, Shenzhen 518118, China; 1810412003@email.szu.edu.cn (L.H.); yezhixiang@sztu.edu.cn (Z.Y.); wudan@sztu.edu.cn (D.W.); 2070413004@stumail.sztu.edu.cn (X.C.); wangning@sztu.edu.cn (N.W.); anhongyu@sztu.edu.cn (H.A.); libobo@sztu.edu.cn (B.L.); xiangbingxi@sztu.edu.cn (B.X.); 2 Guangdong University Key Lab for Advanced Quantum Dot Displays and Lighting, Shenzhen Key Laboratory for Advanced Quantum Dot Displays and Lighting, and Department of Electrical and Electronic Engineering, Southern University of Science and Technology, Shenzhen 518055, China; 11849608@mail.sustech.edu.cn (Z.W.); wangk@sustech.edu.cn (K.W.); 3 Key Laboratory of Optoelectronic Devices and Systems of Ministry of Education and Guangdong Province, College of Physics and Optoelectronic Engineering, Shenzhen University, Shenzhen 518060, China; wangwg@mail.sustech.edu.cn

**Keywords:** perovskite light-emitting diode, FAPb_0.7_Sn_0.3_Br_3_ film, double hole transport structure, light-emitting layer thickness

## Abstract

Highly luminescent FAPb_0.7_Sn_0.3_Br_3_ nanocrystals with an average photoluminescence (PL) quantum yield of 92% were synthesized by the ligand-assisted reprecipitation method. The 41-nm-thick perovskite film with a smooth surface and strong PL intensity was proven to be a suitable luminescent layer for perovskite light-emitting diodes (PeLEDs). Electrical tests indicate that the double hole-transport layers (HTLs) played an important role in improving the electrical-to-optical conversion efficiency of PeLEDs due to their cascade-like level alignment. The PeLED based on poly[(9,9-dioctylfluorenyl-2,7-diyl)-co-(4,40-(N-(p-butylphenyl))-diphenylamine)] (TFB)/poly(9-vinylcarbazole) (PVK) double HTLs produced a high external quantum efficiency (EQE) of 9%, which was improved by approximately 10.9 and 5.14 times when compared with single HTL PVK or the TFB device, respectively. The enhancement of the hole transmission capacity by TFB/PVK double HTLs was confirmed by the hole-only device and was responsible for the dramatic EQE improvement.

## 1. Introduction

Metal halide perovskite nanocrystals (NCs) have received much attention due to their promising applications in optoelectronic devices, such as solar cells, solid-state light-emitting diodes, photodetectors, and lasers [1,2,3]. Excellent or even recorded performance has been achieved due to superior optoelectronic properties, including high photoluminescence quantum yield (PLQY) and charge carrier mobility, narrow emission spectrum, large carrier diffusion length, easily tunable bandgap, and low preparation costs. Therefore, perovskite NC has been regarded as a promising material for next-generation lighting and display applications [4,5]. However, perovskite light-emitting diodes (PeLEDs) have relatively low luminescence efficiency due to the agglomeration of nanocrystalline materials and the imbalance of carrier injection, which hinders their applications in the field of light emission. Recently, various strategies, for example, device interface optimization [6], compositional doping engineering [7,8] and surface ligand engineering [9,10], have been extensively investigated to improve the performance of PeLEDs. The external quantum efficiency (EQE) values of green [11] and red [12] PeLEDs based on submicrometer-scale structures exceed 20%. Although rapid progress has been made in the research of PeLEDs, their luminescence mechanism is still unclear. Moreover, the main shortcomings, such as their poor stability in humid environments, the imbalance between electron and hole injection and the toxicity of lead (Pb), still limit their further practical applications [13].

To overcome these disadvantages, many efforts have been made. A useful way to tune the optoelectronic properties and to reduce the toxicity of Pb in perovskite devices is to mix or partially substitute the A-site cation [14,15]. Usually, some metals, such as Sn, Ge, Fe, Co, Sr, Cu, Zn, and Mn, are used to replace Pb in the perovskite composition to reduce the toxicity of the material and to obtain high-performance PeLEDs [16,17,18,19,20,21,22]. This strategy has been proved to be successful in perovskite PeLEDs [23] and solar cells [1]. Among these alternative metals, Sn has been considered the most suitable substitute of Pb because it has a similar electronic structure and very similar ionic radius. Therefore, the fabrication process and luminescence mechanism of Sn-based (including Sn–Pb) perovskite have been widely investigated recently [24,25,26]. Despite exhibiting excellent light absorption characteristics in solar cells, Sn-Pb perovskites show lower light-emitting properties compared to Sn-free Pb-based perovskites. This is because Sn^2+^ is easily oxidized to Sn^4+^, which leads to the formation of a higher defect density and lower PLQY in LED devices [18,27]. The value of EQE up to 5% for color-pure Sn-based perovskite LEDs has been obtained in recent reports, but its current density remains low and cannot be used in the display field [28]. Therefore, more research should be carried out to further study the carrier transport mechanism and to improve the electro-optical conversion efficiency of Sn-Pb perovskite LEDs.

In this work, we used Sn partially instead of Pb in the perovskite composition to adjust the band gap [16] and to improve crystal quality. By controlling the mole ratio of Sn to Pb, FAPb_0.7_Sn_0.3_Br_3_ nanocrystals with the highest PLQY exceeding 92% can be synthesized at room temperature (RT). The thickness of the FAPb_0.7_Sn_0.3_Br_3_ film was optimized to reduce leakage current and prevent agglomeration of nanocrystals. In order to improve luminous efficiency, we compared the effects of a single PVK hole-transport layer (HTL), single TFB HTL, and double TFB/PVK HTLs on the photoelectric properties of FAPb_0.7_Sn_0.3_Br_3_ PeLEDs. Our results indicate that TFB/PVK double HTLs are useful to realize high-performance in FAPb_0.7_Sn_0.3_Br_3_ films.

## 2. Materials and Methods

### 2.1. Materials

Lead bromide (PbBr_2_, 99.99%), formamidinium bromine (FABr, 99.5%) powder 1,3,5-tris(1-phenyl-1H-benzimidazol-2-yl) benzene (TPBi, 99.99%) and poly [bis(4-phenyl) (2,4,6-trimethylphenyl) amine] (PTAA) were provided by Xian p-OLED. Tin bromide powder (SnBr_2_, 99%), acetonitrile (CH_3_CN, anhydrous, 99.8%), (HBr, 48 wt.% in H_2_O, 99.99%), and N, N-dimethylformamide (C_3_H_7_NO, anhydrous, 99.8%) were purchased from Sigma–Aldrich. Lithium fluoride (LiF, 99.99%), oleic acid (C_18_H_34_O_2_), methylamine (CH_5_N, 30~33 wt.% in ethanol), and oleylamine (C_18_H_37_N, 80~90%) were acquired from Aladdin. The remaining solvents (e.g., toluene, hexane, chloroform, poly [9,9-dioctylfluorene-co-N-[4-(3-methylpropyl)]-diphenylamine] (TFB, Mw > 30,000), poly(N,N0-bis(4-butylphenyl) N, N0-bis(phenyl)benzidine) (Poly-TPD, Mw > 10,000), poly(9-vinylcarbazole) (PVK, Mw > 100,000)), and poly (3,4-ethylene dioxythiophene): polystyrene sulfonicacid (PEDOT:PSS) were obtained from Shanghai Lingfeng Chemical Reagents (Shanghai, China).

### 2.2. Synthesis of FAPb_1-x_Sn_x_Br_3_ NCs

FAPb_1-x_Sn_x_Br_3_ NCs were synthesized at room temperature by the ligand-assisted reprecipitation process (LARP). The synthesis process was divided into three steps: precursor solution preparation, formation of perovskite-suspended solution and nanocrystalline purification, which is slightly different from the method reported in the literature [29]. Firstly, 50 mg SnBr_2_ and a PbBr_2_ mixture with stoichiometric ratios of 0:1, 3:17, 3:7, and 9:11 and 50 mg FABr were dissolved in 1 mL of N, N-dimethylformamide (DMF) solution forming a mixture, and then 200 μL oleic acid and 40 μL oleylamine were injected into the mixture to obtain the perovskite precursors. In order to obtain a homogeneous solution, the mixtures were stirred with a vortex mixer for 1 min. Secondly, after thorough stirring, 400 μL of the perovskite precursors were added to a beaker containing 10 mL of chloroform with magnetic stirring at 12,000 revolutions per minute (rpm). At this time, yellow colloidal solutions appeared, indicating that NCs had formed, resulting in the formation of perovskite-suspended solutions. The final step was nanocrystalline purification: to further purify these solutions, 5 mL of toluene/acetonitrile mixture (volume ratio 1:1) was added to the colloidal solutions and stirred for 1 min. Then, they were transferred to a 50 mL centrifuge tube and dispersed into 2.5 mL n-hexane after centrifuging at 7500 rpm for 4 min.

### 2.3. Fabrication of LED Devices

To further confirm the superior luminescence properties of FAPb_0.7_Sn_0.3_Br_3_ NCs, single HTL and double HTL LED heterojunctions were fabricated with the structure of glass/ITO/PEDOT:PSS (45 nm)/HTL(40~50nm)/FAPb_0.7_Sn_0.3_Br_3_ NCs (41 nm)/TPBi (40 nm)/LiF (1 nm)/Al (100 nm). Before the films were spin coated, ITO glasses with a sheet resistance of 20 Ω/sq were ultrasonically cleaned in detergent, deionized water, alcohol, and isopropanol for 10 min each. In order to increase the adhesion between ITO and PEDOT:PSS, the glass substrates must be treated in ultraviolet–ozone for 20 min. Then, PEDOT:PSS (Clevios AI4083) was spin-coated onto the cleaned ITO glasses with a rotating speed of 3000 rpm for 45 s, followed by heat treatment (bake) in air at 130 °C for 15 min. Afterwards, these abovementioned substrates were transferred to a glovebox filled with N_2_. Next, the HTL solutions were spun onto the top surface of the PEDOT:PSS. For devices with a single HTL of TFB, PTAA or PVK, 8 mg/mL HTL chlorobenzene solution was spin-coated onto PEDOT:PSS at 3000 rpm for 60 s and baked at 120 °C for 20 min to remove the excess chlorobenzene. As for the TFB/PVK double HTL device, firstly, TFB (dissolved in chlorobenzene, 8 mg/mL) was spin-cast with a rotating speed of 3000 rpm for 60 s, followed by annealing at 120 °C for 20 min, and then 2 mg/mL 1,4-dioxane PVK solution was spin-coated onto the TFB layer at 3000 rpm for 45 s and baked at 120 °C for 10 min. As for the light-emitting layer film, FAPb_0.7_Sn_0.3_Br_3_ NC solution (10 mg/mL in n-hexane) was spin-coated with a rotating speed of 1500 rpm for 40 s. Eventually, TPBi (40 nm), LiF (1 nm), and Al (100 nm) were sequentially deposited onto the FAPb_0.7_Sn_0.3_Br_3_ light-emitting layer by a vacuum evaporation method with a growth pressure of 5 × 10^−4^ Torr and a deposited speed of 0.1 nm∙s^−1^.

### 2.4. Characterization

The morphologies and structures of the as-synthesized FAPb_0.7_Sn_0.3_Br_3_ NCs were measured by an FEI Tecnai (Hillsboro, USA) scanning transmission electron microscope (STEM) equipped with an energy-dispersive X-ray spectroscope (EDX, Oxford, UK) and x-ray diffraction (XRD, JEOL, Tokoy, Japan) with a Cu Kα radiation source (λ = 0.1541 nm), respectively. The thicknesses of the as-prepared thin films were characterized by a Bruker Dektak XT Stylus Profiler (Billerica, MA, USA). Steady photoluminescence (PL) and the lifetime decay measurements were performed at room temperature using a Fluorescent Time 300 instrument equipped with a 355 nm excitation source (PICOQUANT, Berlin, Germany). The optical absorption spectra were measured with a Torastsu UV-2600 spectrometer (Milton Keynes, UK). The thin film surface morphologies were examined by atomic force microscopy (AFM, Keysight 5600LS AFM/SPM, Santa Rosa, CA, USA). The forward direction photon-emitted electroluminescence (EL) spectra and the current density–voltage (J–V) characteristics of the prepared LED devices were detected by a fiber optic spectrometer (Ocean Optics USB 2000, Dunedin, FL, USA), a dual-channel Keithley 2614B source meter (Beaverton, OR, USA), and a UDT PIN-25D silicon photodiode (Hawthorne, CA, USA), respectively. The absolute PLQYs of as-prepared NCs were determined by a Quantaurus-QY absolute PLQY spectrometer (HAMAMATSU, Hamamatsu, Japan) using a 350 nm excitation source.

## 3. Results and Discussion

**Figure 1:**
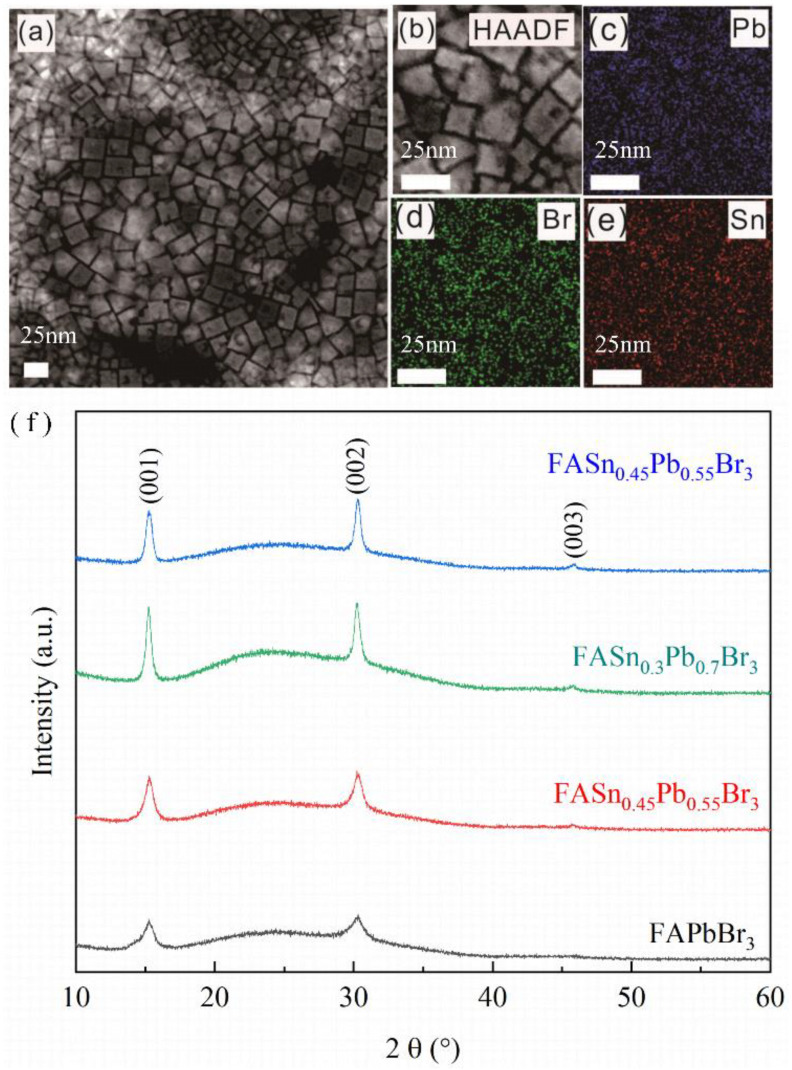
(**a**) TEM image of the FAPb_0.7_Sn_0.3_Br_3_ nanocrystal (**b**) High−angle dark field image of the FAPb_0.7_Sn_0.3_Br_3_ nanocrystal (**c**) Pb (**d**) Br, and (**e**) Sn element mapping results (**f**) XRD patterns of FAPb_1-x_Sn_x_Br_3_ nanocrystalline films with different Pb/Sn ratios.

To investigate the morphologies of the as-prepared FAPb_1-x_Sn_x_Br_3_ NCs, high-angle annular dark field scanning TEM (HAADF-STEM) analyses were carried out (shown in Figure 1). Figure 1a shows that all samples were in the cubic phase with an average size of 20 nm. Figure 1b shows the magnification image of FASn_0.3_Pb_0.7_Br_3_ nanocrystals, and the corresponding element mapping images of Pb, Br, and Sn are shown in Figure 1c–e. Element Sn was observed in the STEM mapping, indicating that Sn was incorporated into these perovskite nanocrystals. With increasing x from 0 to 0.45, the Sn/Pb atom ratio in the FAPb_1-x_Sn_x_Br_3_ NCs was identified to be 0, 16.6, 28.5, and 52.4% (Figure S1a–d and Table S1, Supplementary Materials), which is consistent with the results reported by our cooperating laboratory [8,16]. XRD analysis was also performed to characterize the structure and phase of the as-prepared products. Figure 1f illustrates three diffraction peaks. The peaks centered at 15.25°, 30.3°, and 45.8°can be indexed to the cubic phase FAPb_1-x_Sn_x_Br_3_ (001) and the (002) and (003) crystal planes, respectively. No obvious peak shift or other phases appeared even though the Sn content increased to 0.45, indicating that FAPb_1-x_Sn_x_Br_3_ nanocrystals have purely cubic crystalline structures, which is in accordance with the STEM results. Moreover, compared with other samples, the XRD peak intensity of FAPb_0.7_Sn_0.3_Br_3_ NCs was the highest, and the full width at half maximum (FWHM) of the peak was the narrowest, indicating that its crystallinity was the best. Similar results were also reported in literature 16. In order to avoid repetition, we only focused on FAPb_0.7_Sn_0.3_Br_3_ NCs here. In order to prove that the nanocrystals prepared by the LARP method had good reproducibility, five different batches of FAPb_0.7_Sn_0.3_Br_3_ nanocrystals were synthesized. The average PLQY of all nanomaterials reached 92%, and the test results are shown in the Supplementary Materials (Figure S2a,b).

**Figure 2:**
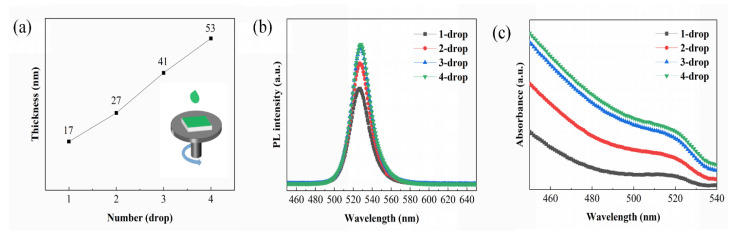
(**a**) The thicknesses of the perovskite layers, (**b**) Photoluminescence spectra, and (**c**) Absorption spectra of the thin films formed by dynamic spin coating of the FAPb_0.7_Sn_0.3_Br_3_ nanocrystalline solution with different dosages of droplets on ITO/PEDOT:PSS/TFB.

The thickness of the perovskite light-emitting layer is critical to the efficiency and stability of the PeLED. Many studies have shown that the optimal thickness of the light-emitting layer should be kept in the range of 35~40 nm because a light-emitting film that is too thick generates more heat (Joules) during the operation of the device, which affects the stability and light coupling efficiency of the device [30]. To explore the effect of the nanocrystalline solution on the thickness and morphology of the light-emitting layer, the dynamic spin coating method was adopted. The perovskite nanocrystals synthesized at room temperature were first dissolved in n-hexane solvent, and then the supernatant was purified by centrifugation to obtain a solution with a concentration of 10 mg/mL. All perovskite NC films were spin-coated on the surface of the TFB hole-transport layer. During the dynamic spin coating process, the substrate was rotated at a rate of 1500 rpm. The thicknesses of perovskite film layers were measured by a step profiler. As shown in Figure 2a, when the amount of perovskite NC solution was increased from one to four drops, the thicknesses of the FAPb_0.7_Sn_0.3_Br_3_ NC films were 17 nm, 27 nm, 41 nm, and 53 nm, respectively. It is clearly seen that the thickness of the light-emitting film increased rapidly with the increase in the number of droplets. However, the thickness of the film did not increase exponentially with the increase in the number of droplets because the subsequent dripping of the solution can wash away part of the underlying nanocrystalline film. To verify the repeatability, five batches of FAPb_0.7_Sn_0.3_ Br_3_ NC films with different numbers of solution drops were tested; the discrepancy of the corresponding film thickness was within 3 nm.

To investigate the effect of the solution dosage on the optical properties of the luminescent films prepared by the dynamic spin coating method, the PL and absorption spectra were measured, and the results are shown in Figure 2b,c. Here, a 355 nm wavelength laser was used as the excitation light. Figure 2b demonstrates the PL spectra of the FAPb_0.7_Sn_0.3_Br_3_ films made using different numbers of drops. One peak centered at 526 nm was observed in these patterns. As the number of droplets increased from 1 to 4, the PL intensity of the perovskite nanocrystalline film increased obviously. This might have been caused by the increase in the number of luminescent particles. When the perovskite film was formed from one drop of the solution, nanocrystals could not completely cover the substrate, and there were some voids between the grains. As the number of droplets increased, the voids were filled, accompanied with the increase in the coverage of nanocrystals, which led to an increase in the PL strength of the perovskite film. In contrast, the PL intensity of the films prepared by 3-drop and 4-drop solutions had little change because the nanocrystals covered the substrate surface. Moreover, for all FAPb_0.7_Sn_0.3_Br_3_ films, the luminous peak positions were constant, and the FWHM of the luminous peaks was still 23 nm, indicating that the agglomeration of nanocrystals was not obvious. From Figure 2c, we can see that the intensity of the absorption spectrum increased gradually as the number of droplets increased. This is reasonable considering that the incident light can be more fully absorbed by the FAPb_0.7_Sn_0.3_Br_3_ film as the thickness of the film increases.

**Figure 3:**
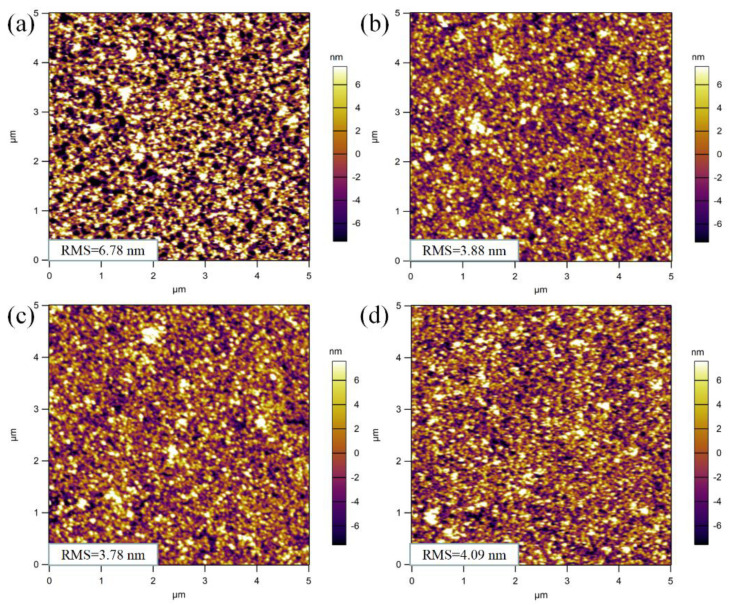
The AFM images of the films (**a**) one drop, (**b**) two drops, (**c**) three drops, and (**d**) four drops formed by dynamic drop−coating of the FAPb_0.7_Sn_0.3_Br_3_ nanocrystalline solution.

The surface morphology of perovskite NC films will affect the performance of PeLEDs, so atomic force microscopy (AFM) was used to directly observe the surface microstructure of the FAPb_0.7_Sn_0.3_Br_3_ NC films; the results are shown in Figure 3. As the number of droplets increased from one to four drops, the surface roughness of these films first decreased obviously and then slightly increased. AFM measurements showed that the root mean square (RMS) roughness of the FAPb_0.7_Sn_0.3_Br_3_ film decreased significantly from 6.78 nm to 3.78 nm with increasing droplet number from one to three drops. However, a further increase in the perovskite solution to four drops yielded a higher RMS roughness of 4.09 nm. The formation of a relative smooth and connected surface morphology for the film prepared from the 3-drop solution was due to the filling of the surface cavities. In contrast, the increase in RMS roughness for the film made from the 4-drop solution was due to a slight surface agglomeration of the FAPb_0.7_Sn_0.3_Br_3_ nanocrystals. Therefore, combining the results of surface roughness (shown in Figure 3c) and PL spectra (shown in Figure 2b), the optimal thickness of the light-emitting layer film was 41 nm, similar to the results obtained in [16,30].

**Figure 4:**
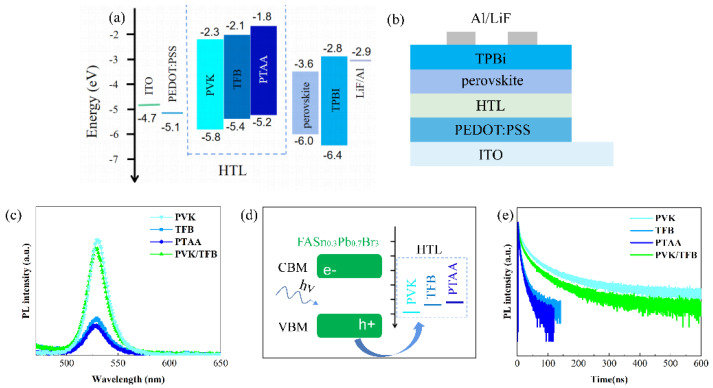
(**a**) Energy−level diagram and (**b**) schematic diagram of single HTL PeLEDs. (**c**) PL intensities, (**d**) Charge transfer model and (**e**) Transient photoluminescence (TRPL) spectra of the FAPb_0.7_Sn_0.3_Br_3_ perovskite films coated on the top of PVK, TFB, PTAA, and TFB/PVK, respectively.

In addition to the influence of the light-emitting film, the hole-transport layer also plays an important role in the performance of the PeLED [9]. Before the preparation of devices, we compared the performance of different HTLs in detail. The PVK, TFB, and PTAA are often used as the HTLs of PeLEDs [9,31,32]. Compared with the valence band top (−6.0 eV) for FAPb_0.7_Sn_0.3_Br_3_ NC materials, the highest occupied molecular orbital (HOMO) energy level of PTAA (−5.2 eV) was relatively shallow, which makes it difficult for holes to be injected into the FAPb_0.7_Sn_0.3_Br_3_ light-emitting layer [9]. Some researchers used TFB instead of PTAA as a PeLED hole injection layer to enhance the hole transport ability and the device performance because TFB has a deeper HOMO level (−5.4 eV) and a higher hole mobility (1 × 10^−2^ cm^2^ V^−1^ s^−1^) [33] than PTAA (−5.2 eV, 5 × 10^−3^ cm^2^ V^−1^ s^−1^) [34]. However, in organic LED devices, PVK is the most commonly used HTL material because its HOMO energy level (−5.8 eV) is deep. This means that the hole is easily injected into the perovskite light-emitting layer due to the small energy barrier between the HTL and the FA Pb_0.7_Sn_0.3_Br_3_ NC layer (−6.0 eV). The energy level diagrams of various components in the PeLED structures and schematic diagrams of the PeLED device structure are shown in Figure 4a,b.

Figure 4c shows that the optical properties of FAPb_0.7_Sn_0.3_Br_3_ NCs exhibited strong dependence on the underlayer films. The perovskite NCs had the highest PL value and a slight fluorescence quenching on the hole-transport layer of PVK, whereas the FA Pb_0.7_Sn_0.3_Br_3_ film coated on the TFB showed a significant decrease in luminous intensity. In contrast, the PTAA hole-transport layer resulted in the most severe fluorescence quenching of perovskite NCs. This marked fluorescence quenching may relate to the energy gaps (ΔEs) between the VBM of FAPb_0.7_Sn_0.3_Br_3_ and the HOMO levels of the HTLs. Figure 4a illustrates that the ΔEs between the NCs and the PVK, TFB, and PTAA were 0.2 eV, 0.6 eV, and 0.8 eV, respectively. Hence, for FAPb_0.7_Sn_0.3_Br_3_ nanocrystals on PVK, the smallest energy level difference (0.2 eV) was the main reason for the smallest fluorescence quenching and a higher hole extraction of thin films. Compared with the single HTL, The PL intensity of the perovskite film coated on the TFB/PVK double HTLs was significantly improved. Cascade-like energy alignment caused by the PVK as a transition layer between the TFB and the FAPb_0.7_Sn_0.3_Br_3_ film was responsible for the enhancement of the PL peak. The photogenerated carriers generated by the perovskite layer were quickly transferred to the adjacent PVK hole-transport layer due to the smaller ΔE, which led to a large number of holes in the devices. Similar conclusions have been verified in solar cells [35]. The schematic diagram of hole transfers between the perovskite film and the HTLs is shown in Figure 4d.

Transient photoluminescence (TRPL) decay measurements were carried out at room temperature to further study the dynamic recombination of carriers in the FAPb_0.7_Sn_0.3_Br_3_ films on different HTLs. Figure 4e demonstrates the TRPL decay curves. The average effective PL lifetimes of FAPb_0.7_Sn_0.3_Br_3_ nanocrystal films on different transmission layers (PVK, TFB, PTAA, and PVK/TFB) were 46.5 ns, 5.4 ns, 2.5 ns, and 28.5 ns, respectively, which is agreement with the PL results shown in Figure 4c. This dramatic decrease in the average effective PL lifetimes when the HTL layer changed from PVK to PTAA can be caused by the increase in non-radiative recombination due to the defect status [23]. A similar increase in the nonradiative deexcitation path was also proposed for Sn-doped perovskite film [20]. However, compared with the average PL lifetime of perovskite film on TFB (5.4 ns), the PL lifetime of the FAPb_0.7_Sn_0.3_Br_3_ film with TFB /PVK double HTLs and PL intensity increased obviously (Figure 4c). These increases in both luminescence lifetime and luminescence intensity are attributed to the reduction of non-radiative recombination pathways by inserting the PVK layer between the TFB and the perovskite layer, which can help improve the performance of the PeLED devices.

**Figure 5:**
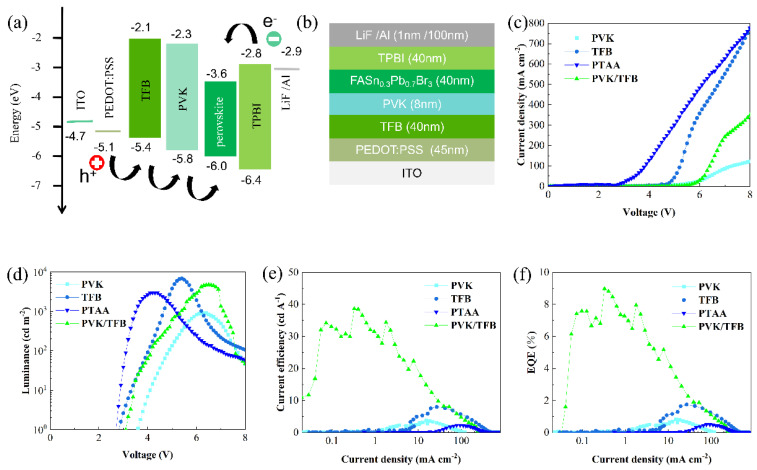
The performance curves of the devices based on single HTL of PVK, TFB, PTAA and double TFB/PVK HTL devices. (**a**) The energy level diagram of TFB/PVK HTL PeLED and (**b**) device configuration of the as-fabricated PeLED based on double HTLs. (**c**) Current density−voltage, (**d**) Luminance−voltage, (**e**) EQE−current density, and (**f**) Current efficiency−current density characteristics of FAPb_0.7_Sn_0.3_Br_3_ NC PeLEDs.

In order to prove the luminescence characteristics of perovskite nanocrystals and study the effect of different hole layers on device performance, we fabricated single and a double HTL PeLEDs with the structure of glass/ITO/PEDOT:PSS (45 nm)/HTL/FAPb_0.7_Sn_0.3_Br_3_ NCs (41 nm)/TPBi (40 nm)//LiF (1 nm)/Al (100 nm). The corresponding single HTL and double HTL PeLED schematic diagrams are shown in Figure 4b and Figure 5b, respectively. Herein, PEDOT:PSS is the hole injection layer, PVK is hole-transport layer, perovskite FAPb_0.7_Sn_0.3_Br_3_ NC film is the light-emitting layer, and TPBi is used as the electron transport layer. In addition, the characteristics of charge density(J)–voltage (V) for the PeLEDs with different HTLs are shown in Figure 5c. The current density of the device with single PVK HTL is the lowest at the high voltage as compared to that of the PeLEDs with TFB, PTAA and TFB / PVK HTLs. Two reasons may be responsible for the low current density of the PVK device. One is that mobility of PVK (10^−6^ cm^2^ V^−1^ s^−1^) [9] is much smaller than that of TFB (1 × 10^−2^ cm^2^ V^−1^ s^−1^) and PTAA (5 × 10^−3^ cm^2^ V^−1^ s^−1^), so the injection of holes in the device is slow. Moreover, the electron mobility of TPBi (10^−4^ cm^2^ V^−1^ s^−1^) [36] is much higher than the hole mobility of PVK HTL, which leads to an imbalance in electron and hole injection. The other is the large hole-injection barrier between the HOMO level of PEDOT:PSS (−5.1 eV) and the PVK (−5.8 eV), which blocks hole injection into the perovskite layer. The luminance (L)–voltage (V) distributions of the PeLEDs are shown in Figure 5d. Compared to devices using TFB and PTAA as HTL, the device with single PVK HTL exhibits a higher turn-on voltage (≈3.6 V) because of its weak hole-transport ability as mentioned above. The EQE of 1.75% for the devices with single TFB HTL is higher than that of the devices with PVK (0.82%) and PTAA (0.5%) HTLs. The relatively high EQE and current efficiency (7.8 cd/A, shown in Figure 5f) for the single TFB device is attributed to fast hole migration. Combining the results of PL (shown in Figure 4c), we conclude that the electroluminescence performance of the FAPb_0.7_Sn_0.3_Br_3_ PeLED is not only related to the fluorescence lifetime of quantum dots but also to the mobility of holes.

Compared with single hole transport devices, PeLED fabricated with the TFB/PVK double hole transport structure exhibited better electroluminescence performance due to the formation of a cascade-like level alignment, which facilitates hole transport (shown in Figure 5a) [9]. The TFB/PVK double HTLs were constructed by coating a layer of PVK (8 nm) film on the surface of the TFB film (40 m), and the film thickness of each layer was measured by a step meter. As illustrated in Figure 5e,f, the maximal EQE was found to be 9% in the TFB/PVK double HTL device with a current efficiency peak value of 38.6 cd A^−1^ and a turn-on voltage of 3.2 V (shown in Figure 5d), which was improved by approximately 10.9 and 5.14 times as compared to the single HTL PVK or TFB device, respectively. The formation of a cascaded energy band structure by inserting TFB (−5.4 eV) between PEDOT:PSS (−5.1 eV) and PVK (−5.8 eV) in the double HTL device might be the main reason for the significant improvement in the electroluminescent properties. Therefore, we demonstrate that the TFB/PVK double HTL structure is favorable for hole injection (shown in Figure 5a), which is an effective approach to improve the PeLED performance.

**Figure 6:**
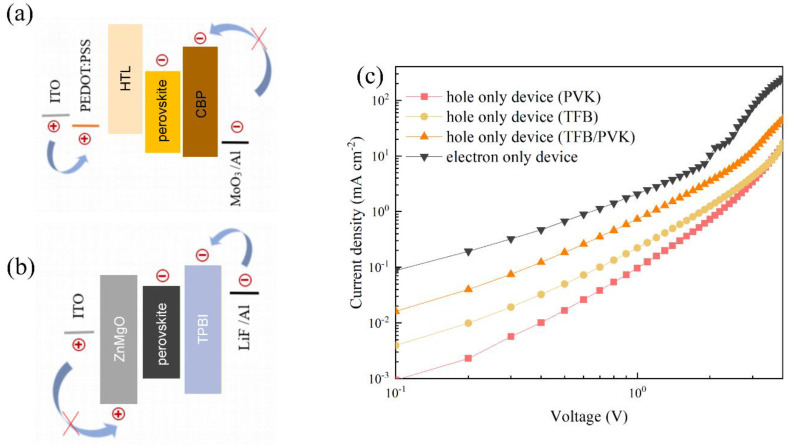
(**a**) Energy level structure diagram of a hole−only device with different hole−transport layers (**b**) Energy level structure diagram of an electron−only device with TPBi as the electron transport layer (**c**) J−V curves of the hole−only and electron−only devices with TFB, PVK, and TFB/PVK HTLs.

In order to confirm the above deduction and to investigate the effect of single/double HTLs on charge injection, the electron-only device (EOD) and the hole-only devices (HODs) were fabricated with the structure of ITO/ZnMgO/FAPb_0.7_Sn_0.3_Br_3_/TPBi/LiF/Al and ITO/PEDOT:PSS/HTL/FAPb_0.7_Sn_0.3_Br_3_/CBP/MoO_3_/AL, respectively. The alignment of energy levels of the electron-only device and hole-only devices based on PVK, TFB, and TFB/PVK HTLs is shown in Figure 6a,b. Figure 6c shows that the hole current density of HOD based on the TFB/PVK double layer structure was much higher than that of the HODs with a single PVK or TFB HTL, indicating that double HTLs are a feasible approach to enhance the current density of holes. At the same time, we noticed that the current density of EOD was higher than that of HOD, implying that the injection rate of electrons was much faster than that of holes. This led to electrons becoming the main carrier in our constructed devices. However, among the three HOD devices, the current density of HOD with the TFB/PVK structure was the highest, which was most similar to that of EOD. These results demonstrate that TFB/PVK double HTLs can facilitate hole injection and promote charge balance, consistent with the higher EQE shown in Figure 5f.

## 4. Conclusions

In summary, we synthesized highly luminescent FAPb_0.7_Sn_0.3_Br_3_ nanocrystals with an average photoluminescence quantum yield of 92%. The luminescent layer film with 41 nm thickness, which has a relatively smooth surface and a strong PL peak, was prepared by the dynamic spin-coating method. The electrical properties revealed strong dependence on the hole-transport material. The maximal external quantum efficiency of 9% and the current efficiency of 38.6 cd∙A^−1^ were obtained in the FAPb_0.7_Sn_0.3_Br_3_ PeLED with TFB/PVK double HTLs. Compared with devices based on PVK and TFB single HTL, the EQE of the double HTLs device was improved by approximately 10.9 and 5.14 times, respectively. This improvement can stem from the enhancement of the hole transmission capacity by TFB/PVK double HTLs in FAPb_0.7_Sn_0.3_Br_3_ PeLEDs. The hole-only device confirms that the formation of the cascade level alignment through a double hole transport structure is an effective approach to promote the hole injection and charge balance. Our findings provide an approach to improve the luminous efficiency of light-emitting devices with a large energy gap between two adjacent layers.

## Data Availability

The data presented in this study are available on request from the corresponding author.

## Supplementary Materials

The following supporting information can be downloaded from https://www.mdpi.com/article/10.3390/nano12091454/s1, Figure S1: EDX analysis of the FAPb_1−x_Sn_x_Br_3_ nanocrystals with different Sn contents (a) x = 0, (b) x = 0.15, (c) x = 0.3, (d) x = 0.45; Figure S2: FASn_0.3_Pb_0.7_Br_3_ nanocrystals (a) under ambient light irradiation, (b) under ultraviolet light irradiation, (c) PLQY statistics of as-prepared nanocrystals synthesized in different batches; Table S1: Atomic ratio of Sn to Pb in FAPb_1−x_Sn_x_Br_3_ nanocrystals measured by EDX.
