# Supplementary material for: Marked Efficiency Improvement of FAPb0.7Sn0.3Br3 Perovskite Light-Emitting Diodes by Optimization of the Light-Emitting Layer and Hole-Transport Layer
Source: Nanomaterials (Basel). 2022 Apr 25;12(9):1454. doi: 10.3390/nano12091454 (PMC9102924; doi:10.3390/nano12091454)
Supplement: Supplementary file 1 [file nanomaterials-12-01454-s001.zip › nanomaterials-1669132-supplementary.pdf]

nanomaterials

MDPI

Supplementary Materials

Marked Efficiency Improvement of $FAPb_{0.7}Sn_{0.3}Br_3$ Perovskite Light-Emitting Diodes by Optimization of the Light-Emitting Layer and Hole-Transport Layer

Lufeng Hu [1,†], Zhixiang Ye [1,†], Dan Wu [1], Zhaojin Wang [2], Weigao Wang [3], Kai Wang [2], Xiangqian Cui [1], Ning Wang [1], Hongyu An [1], Bobo Li [1], Bingxi Xiang [1] and Mingxia Qiu [1,*]

[1] College of New Materials and New Energies, Shenzhen Technology University, Shenzhen, Guangdong, 518118, China; 1810412003@email.szu.edu.cn (L.H.); yezhixiang@sztu.edu.cn (Z.Y.); wudan@sztu.edu.cn (D.W.); 2070413004@stumail.sztu.edu.cn (X.C.); wangning@sztu.edu.cn (N.W.); anhongyu@sztu.edu.cn (H.A.); libobo@sztu.edu.cn (B.L.); xiangbingxi@sztu.edu.cn (B.X.)

[2] Guangdong University Key Lab for Advanced Quantum Dot Displays and Lighting, Shenzhen Key Laboratory for Advanced Quantum Dot Displays and Lighting, and Department of Electrical and Electronic Engineering, Southern University of Science and Technology, Shenzhen, Guangdong, 518055, China; 11849608@mail.sustech.edu.cn (Z.W.); wangk@sustech.edu.cn (K.W.)

[3] Key Laboratory of Optoelectronic Devices and Systems of Ministry of Education and Guangdong Province, College of Physics and Optoelectronic Engineering, Shenzhen University, Shenzhen, Guangdong 518060, China; wangwg@mail.sustech.edu.cn

* Correspondence: qiumingxia@sztu.edu.cn

† These authors contributed equally to this work.

Energy dispersive X-ray spectroscopy (EDX) of $FAPb_{1-x}Sn_xBr_3$ nanocrystals with different Pb/Sn ratios was performed, and the results shown in Figure S1a–d. Peaks of Pb, Sn and Br are shown in the EDX spectra, indicating these components make up $FAPb_{1-x}Sn_xBr_3$ quantum dots. As increasing x from 0 to 0.45, the Sn doping atom ratios were found to be 0 (x = 0), 16.6% (x = 0.15), 28.5% (x = 0.3), and 52.4% (x = 0.45), respectively, which is consistent with the results reported in our cooperating laboratory [8,16]. The test results are shown in Table S1. The content of Sn in the $FAPb_{0.55}Sn_{0.45}Br_3$ nanocrystals exceeds the doping value of 0.45, which is caused by the surface precipitation of Sn element.

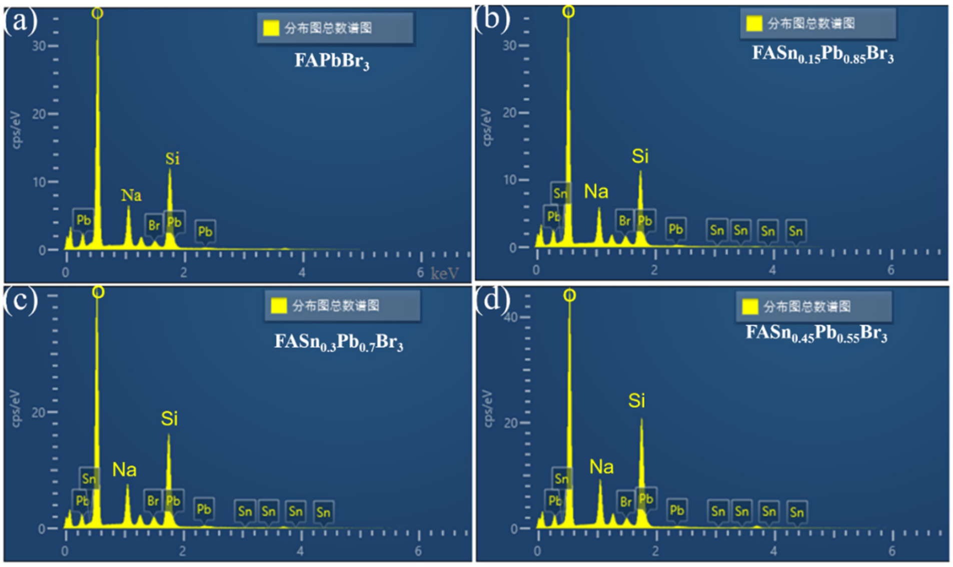

Figure S1. EDX analysis of the $FAPb_{1-x}Sn_xBr_3$ nanocrystals with different Sn contents (**a**) x = 0, (**b**) x = 0.15, (**c**) x = 0.3, (**d**) x = 0.45.

Table S1. Atomic ratio of Sn to Pb in $FAPb_{1-x}Sn_xBr_3$ nanocrystals measured by EDX.

$FAPb_{1-x}Sn_xBr_3$	Source Materials Atom Ratio $SnBr_2$:,$PbBr_2$	Film Sn/Pb Ratio, at%
X = 0	0:1	0
X = 0.15	3:17	16.6%
X = 0.3	3:7	28.5%
X = 0.45	9:11	52.4%

Figure S2 illustrates the optical properties of the $FASn_{0.3}Pb_{0.7}Br_3$ nanocrystals measured at room temperature. Figure 2a,b are the sample images under ambient light and ultraviolet light, respectively. It can be seen that the perovskite solution is clear, transparent and can emit green fluorescence under ultraviolet light. In order to prove that the nanocrystals prepared by the LARP method have good reproducibility, five different batches of $FASn_{0.3}Pb_{0.7}Br_3$ nanocrystals were synthesized. The average PLQY of all nanomaterials can be as high as 92%, and the test results are shown in Figure S2a,b.

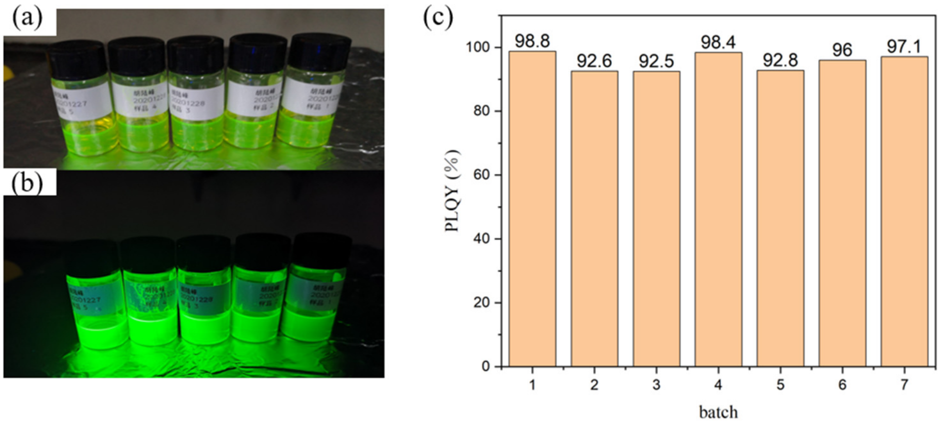

Figure S2. $FASn_{0.3}Pb_{0.7}Br_3$ nanocrystals (**a**) under ambient light irradiation, (**b**) under ultraviolet light irradiation, (**c**) PLQY statistics of as-prepared nanocrystals synthesized in different batches.
